# A very rare case of multifocal non-familial cardiac myxoma involving Left Atrium and Mitral valve necessitating mitral valve replacement along with excision of myxoma

**DOI:** 10.1186/1749-8090-10-S1-A6

**Published:** 2015-12-16

**Authors:** Pai R Krishnanand, Abilash Sathyanarayanan, C Ganesan, Uma G Maheswari, V Chaitra, MS Murugan, Prasanth A Biradhar, PR Murugesan

**Affiliations:** 1 Department of Cardiothoracic and Vascular Surgery, PSG Institute of Medical Sciences and Research, Coimbatore, India; 2 PSG Institute of Medical Sciences and Research, Coimbatore, India; 3 Department of Anaesthesiology, PSG Institute of Medical Sciences and Research, Coimbatore, India; 4 Department of Pathology, PSG Institute of Medical Sciences and Research, Coimbatore, India

## Background/Introduction

Left Atrium [LA] is the commonest and heart valves the least common site for cardiac myxomas. Non-familial multifocal myxomas involving LA and Mitral valve are rare. Only a couple of cases of Excision of LA myxoma with concurrent Mitral valve interventions have been reported in history.

## Aims/Objectives

We report the first case of non-familial myxoma involving left atrium and mitral valve necessitating a MV replacement at time of myxoma excision.

## Method

Echocardiography in a 56 year male, with no family history of cardiac surgery, and presenting with shortness of breath of 3 months revealed a LA myxoma protruding into left ventricle with associated hemodynamically mild Mitral stenosis and moderate Mitral regurgitation. The Mitral valve apparatus and chamber sizes were normal. Hemogram, biochemistry, and liver function tests were normal.

## Results

The myxoma was approached via inter atrial septal and excised. Initial examination of Mitral valve apparatus revealed good leaflet coaptation no annular widening and therefore no interventions were performed. Weaning of bypass was difficult and trans-esophageal echocardiography revealed more than moderate Mitral regurgitation with central jet. The Mitral valve was approached through the Waterston's groove and replaced with a St Jude mechanical prosthesis. Patient was weaned off bypass with stiff ionotropes and recovered uneventfully. Histopathology of excised valve revealed foci of myxoma with superimposed degenerative changes.

## Discussion/Conclusion

We present this interesting case report with images, echocardiography images and discussion on the dilemmas in decision making faced during the procedure, leading ultimately to a favourable outcome.

## Consent

Written informed consent was obtained from the patient for publication of this abstract and any accompanying images. A copy of the written consent is available for review by the Editor of this journal.

